# Cases of Placenta Prævia

**Published:** 1861

**Authors:** Jonathan W. Brooks

**Affiliations:** Chicago, Ills.


### CASES OF PLACENTA PRÆVIA.

By JONATHAN W. BROOKS, M. D., of Chicago, Ills.

The following monograph of five cases of Placenta Prævia, which have occurred under the writer's observation during the last twenty-five years, will perhaps go directly to confirm an opinion expressed by some eminent practitioners, to wit: that the placenta should be entirely separated from the uterus, as early as possible after labor commences, attended by hæmorrhage arising from this peculiar point of attachment; for this important reason, that the hæmorrhage is thereby promptly arrested, and the risks both to mother and child materially lessened.

Case 1st—Was called to L——a M——n, of N—— H——, prima para, unmarried, May 7th, 1836, at 5 o'clock A. M. The following is her own brief and sorrowful history, as narrated to me by Mrs. B., with whom she boarded, her own sister and another relative: An orphan, aged 17 years and 4 months, unmarried; enciente, by her sister's husband. Was drugged constantly for seven months after the interruption of her catamenia, which, from her statement, occurred early in August, 1835. Had never known a sick day until about the tenth of Sept., same year, when she took essential oil of savin in twenty drop doses, three times per diem, for five consecutive days; said it purged and vomited her dreadfully and made her have some show, yet did not remove her burthen, but left a tenderness all over the stomach. From that time to early in April, 1836, she took nostrums and drugs, always being poorly. About April 6th she had some intimations of approaching labor, by cutting pains, &c., accompanied by hæmorrhage; at which time she had a midwife in attendance.

Toward the close of April she was removed from N—— H——, to near my residence for the purpose of concealment. She was then placed in charge of a professed midwife—a stranger to all parties; who, with those in attendance, reported labor as having commenced about four hours previous to my arrival and flooding about two hours. They were not alarmed until half-past four o'clock, when a severe and protracted pain was accompanied by copious flooding and alarming prostration. At 5 o'clock A. M., the following aspect presented itself: The bed and bedding were drenched in blood; the patient restless, her countenance pale and anxious; pulse one hundred and fifteen, quick, and her extremities cold. Examination per vaginam, os uteri very tender, dilatable to some extent, but not well relaxed; placenta placed directly over it; about one-third of that portion towards the left acetabulum of the patient was detached from the uterus, whence was issuing the blood. The membranes were evidently unbroken. It was my first case of the kind.

The danger being imminent, proceeded instantly to detach the residue of the placenta with the index finger of the right hand, which being accomplished, the hæmorrhage ceased, and within six minutes a very heavy, strong pain expelled the placenta, the liquor amnii, and brought the child near the vulva. A second pain followed almost without interval, expelling a well developed boy weighing nine pounds, feeble yet viable. No blood was believed to have been lost after the separation of the placenta. The uterus contracted feebly. The child was handed to an attendant, placenta and all, and afterwards attended to; and was a fine healthy man in May, 1861. A bandage was applied* (of the pattern still preferred

* The bandage consists of a body and four straps, all made double of heavy new muslin, that has been thoroughly softened by washing. Length of body thirty-six inches, width of ends nine inches; the centre should be four inches wider, and that part nine inches between the corners. The point between the widest and narrowest part of the body should form an arc. Two straps, twenty inches long, to pass around the body, five and a half inches wide where they attach to the middle of the body and four and a half inches at the other end. Five inches from the left end of the body should be a perpendicular opening to by me) as speedily as possible to the mother. There was jactitation and constant throwing of her arms and hands, a blanched and still more anxious countenance, pulse one hundred and fifty with intermissions, faintness. Gave Tinct Opii thirty drops, brandy, as much as she could swallow. In thirty minutes repeated the Tinct Opii. In forty minutes, no rallying; gave Tinct. Opii fifteen drops, Spts. Camphor twenty-five drops. In ten minutes more gave six grains Carbonate of Ammonia in a solution of Gum Arabic; applied dry warmth to extremities, &c. But all to no purpose. She continued to sink, and died from exhaustion in about two hours from the birth of the child.

Leave being obtained, an autopsy was had thirty hours after death. External aspect, complexion fair, surface peculiarly white. An indentation on the right parietal bone, a little below the protubuance, said by her friends to have been produced by a fall during a convulsion, which was supposed to be caused by some drug she had taken. On the right side of the abdomen, on a line with the umbilicus, was the appearance of a bruise, dating back some time, and believed by her friends to have occurred at the same time as the indentation of skull. Body very plump; rigor mortis rather slight. On opening the abdomen the intestines and omentum were found agglutinated to each other by inflammatory lymph on exudation. Opposite the bruised point the exudation was more striking. Some blood in the vessels. Portions of the peritonum gave evidence of inflammatory action. Uterus well contracted, more pale than usual; indubitable evidence of the exact attachment of the placenta over the os uteri. The other visceræ of the abdomen in a generally healthy condition, as well as those of the chest. Under the indentation of the cranium was a small extravasation of blood apparently in receive the right strap, which prevents slipping. These straps fasten in front. Two straps, eighteen inches long, one and a half wide, one end of each attached to the body of the bandage, eight inches from each end, and the other ends fastened to the corners of the widest part of the bandage. These embrace the thighs and keep the lower part in situ.

process of removal by absorption; otherwise the contents of the cranium appeared healthy, but pale.

This was a case where ecbolics had been used, so for as we may credit the testimony of others, to a greater extent than has ever fallen under the writer's observation, and, in his opinion, contributed largely to the fatal result.

Case 2nd—E—— M——r, married, has had syphilis and miscarried from the third to the fifth month four times previous to Jan., 1844. Was called to her January 4th, 1745, at 1 o'clock P. M. Examination per vaginam elicited the following condition: Blood oozing freely from os uteri, some tenderness of same.

On interrogation, says she was washing, and while at the tub something seemed to give way and she began to flow hard then, and it still continued, uneasiness and pain in the uterine region. Absolute rest in the recumbent posture, Tinct. Opii twenty drops in water, quiet to be observed. Two o'clock, uneasiness, abating hæmorrhage also. Tinct. Opii, fifteen drops in aqua. At half-past two she fell asleep and continued so for forty minutes; awoke refreshed, with hæmorrhage very slight. Gave ten drops of Tinct. Opii. She soon recovered.

Feb. 8th, 1845—Was summoned to her at 2 o'clock A. M. On arrival the following condition presented itself: Bedding and clothing very much saturated with blood. The patient did not seem exhausted. Reported herself as having had labor pains for two hours. At twenty minutes of two, she had a brisk pain, and flowed, she thinks, ten minutes; then, with another brisk pain, something seemed to come low down in the passage; since which she is confident she has lost no blood.

Examination.—Placenta at the vulva. In ten minutes it was expelled with some liquor amni.

Farther examination informed us that the fœtus was presenting by the feet. In eleven minutes it was expelled by natural efforts, and gave some equivocal signs of viability. The mother believed it to be an eight month's one, and appearances would confirm that opinion. Its abdomen was dropsical. She made a good recovery, and in ten months gave birth to a son at eight months that lived five weeks.

Case 3rd—Was called in consultation in case of Mrs. E—— C——h, May 24th, 1850, with Dr. T——, the attending physician, at 6 o'clock A. M. Believes herself about three months advanced in her sixth pregnancy. Is a pale, delicate woman, of fine intellectual endowments and culture. In her first labor had several convulsions. Complains of pain and weight lower in the maternal organs than she has ever experienced in previous instances. She has for several days had moderate hæmorrhage (passive), but kept up with her family, attending to her ordinary duties. Examination revealed closed os uteri and oozing of blood. Has some headache in the erect posture, with a blowing sound in her ears, which is somewhat relieved by reclining. Pulse ninety, quick and feeble. Heart easily excited to palpitation by noise; bowels relaxed, urine excessive in quantity and pale, feet a little swelled. Absolute rest in recumbent posture. Take Tinct. Opii fifteen drops.

℞ Quinine Sulph. iss℈
Gum Opii Pulv. gr. ivss Mft. Mass.
Syrup Simp q. s. Pillulæ No. xii.

Give one every two hours. If hæmorrhage essentially abates or suspends, once in four hours, generous diet. At 7 P. M. saw her again, by request of her medical adviser. Hæmorrhage abating, free from headache; dined at 1 P. M., on mutton-chop, with roast potatoes and a cup of good Mocha coffee, and has felt better for it. Continue pills once in four hours.

25th, 10 A. M.—Met Dr. T. agreeably to appointment. She had scrupulously followed directions. Head clear, free from pain; has rested well through the night, which she has not done for some weeks previously. To use her expression, she has been fidgetty, wakeful and restless these six weeks past. Bowels moved naturally this morning; urine less in quantity, more color; hæmorrhage inconsiderable; pulse eighty-four, more full and soft; heart's action less easily excited; less uneasiness in the maternal organs. Continue treatment and diet.

26th, 10 A. M.—Met again. The patient still farther improved; very little oozing of blood from os uteri. Expresses herself as feeling better than for two months. Continue pills once in six hours. Still keeping her bed. Suggested to my medical friend that it might be a case of Placenta Prævia.

27th—Still improving; hæmorrhage entirely ceased; continue diet; recline most of the time for several days.

℞ Quinine Sulph. i ℈
Syrup Gum Arabic qs
M. Mass. Pillulae No. xx

Take one every eight hours. From this time her physician informs me, she progressed well, and took no more medicine after exhausting the last prescription, and did not see her again until summoned to meet her medical attendant in consultation, Sept. 25th, at 1 P. M., serious hæmorrhage having supervened, as she thought, by lifting too much an hour previous. On arrival, found her much as on May 24th, except the hæmorrhage was more active and she restless, with frequent sighings; nausea; no vomiting; pulse 110. Examination—Os uteri rigid; nothing like dilatation; tender to the touch; blood oozing freely; some cutting pain and aching in maternal organs; extremities cold; apply warm, dry flannels to them.

℞ Tinct. Opii xxv gtts
Spts. Camphor xx gtts
Sacch Saturni v grs
Aquae q s Haustus.

Half-past two, slightly improved. Repeat draught. Four o'clock, repeat draught. Six, has slept quietly fifty minutes; hæmorrhage greatly abated; wishes food, and partakes of broiled steak and a cup of well dressed coffee, and enjoys them. Half-past eight, still better; repeat draught. Ten, hæmorrhage slight; quiet; cutting pains absent; aching in maternal organs greatly relieved.

℞ Quinine Sulph. ii ℈
Gum Opii V grs
Syrup Gum Arabic qs
M. Mass. Pillulae No. xxv

To be taken one every five hours, commencing at 12 o'clock M.; generous diet.

26th—Better; hæmorrhage suspended; improved in every particular. Pills to be given once in eight hours, No. i. Continue diet and *rest absolute.*

27th—Convalescing. Suggest watchfulness as to hæmorrhage. Am confident it is a case of Placenta Prævia.

Oct. 20th—Called with my friend at 5 P. M. Examination—Some oozing of blood; os uteri rigid. Patient expresses herself, positively, that she thinks it was induced by walking more than usual, and slipping while walking. Is confident she has been better the last three weeks than at any time for the last three months preceding. Her appearance indicates all she expresses. Complains of some uneasiness in the maternal organs; not much tenderness of os uteri; eats well; sleeps well; pulse eighty, nearly natural for fulness and softness; nervousness slight; bowels in good state; urine, she thinks, is about as it is when she is in ordinary good health.

℞ Tinct. Opii x gtts
Aqua q s Haustus.

Repeat once in two hours, until hæmorrhage is arrested. Recumbent posture; quiet.

21st—Better; hæmorrhage entirely ceased; expresses herself as feeling as well as she did three days before she slipped. Suspend medicaments; continue reclining two or three days.

Nov. 22nd—At special request of my excellent friend, was summoned with him at 3 A. M. Examination, on arrival, elicited the fact that the external organs were relaxing; the os uteri was not dilated. There were irregular premonitory pains. Says she feels well; is in good spirits. Her attending physician says he never saw her look better when labor was near. About five o'clock she had a smart labor pain. Examination disclosed the os relaxing and dilating some hæmorrhage; the edge of the placenta loose, presenting. The finger, passing by the loosened edge, came in contact with the membranes unbroken. From this time labor pains steadily increased, and the os relaxing till near seven o'clock, when a brisk pain was attended with a rapid flow of blood. Membranes yet unruptured; the placenta attached towards the right acetabulum of the mother rather anteriorly; blood continuing to flow. Suggested the immediate detaching of the placenta in its entirety, which was accomplished with some difficulty, by the index finger of the right hand; the membranes giving way at the same time, and the head advancing in the first natural position of Dewees. In less than five minutes the placenta was expelled, almost instantly being followed by a female child, weighing six and a half pounds. It moved a little spasmodically at first, gasping occasionally. Soon, by dashing small quantities of cold water on its face and on its chest, and using artificial aspiration by application of mouth to mouth, thus inflating, and then, with the hands, compressing the chest, respiration was fully established, and is now, 1861, a fine healthy girl. Very little blood was lost after the final separation of the placenta from uterus. The mother made an excellent recovery, and has since given birth to one son and three daughters, without complication.

Case 4th—Was called to Mrs. F——, May 22nd, 1856, whose history to that time is briefly this: Complexion, a brunette; married at eighteen years; has uniformly enjoyed good health to May 16th, and is enceinte for the fourth time. The first birth, thirteen months after marriage; the others at intervals of two years; the youngest now a little more than two years old. May 16th, 1856, she fell, injuring her back. Thinks herself about two months advanced in pregnancy.

Has uterine hæmorrhage, which commenced three days since. Reports having had something like her catamenia a month ago, much less in quantity and of a brighter color than natural ; at the same time had the symptoms which invariably attend her during the first months of this state. Examination—Os uteri closed, more full and round than it is generally at the second month, perhaps fairly increased by one-fifth from normal condition ; blood oozing from os uteri. Sensation through the loins, thighs and lower extremities, like those she is accustomed to at the month ; bowels well opened ; urine small in quantity ; pulse eighty, a little full and tense ; face slightly flushed. Diagnosed a case of Placenta Prævia. Venesection six ounces, Spts. Nit Dulcis thirty drops, once in two hours ; absolute rest in recumbent posture, hard bed, care and quiet strictly enjoined ; diet toast and black tea, the latter cool.

23rd—Hæmorrhage suspended. Expresses herself as feeling better decidedly every way. Enjoin rest and quiet with moderate diet for a few days, with injunction to notify me at the earliest possible moment if hæmorrhage returned, which directions were carefully observed.

June 21st, 10 o'clock A. M.—Called again. Patient has been well as usual since May 24th. At nine o'clock, when in the act of rising from her chair and walking across the room, hæmorrhage commenced more brisk than before. She reclined immediately, and sent a messenger to make this announcement. On arrival, instituted an examination.—Found more fullness than is common at this period of pregnancy of the os and cervix uteri ; no dilatation ; the oozing more brisk than a month ago ; has some of the feelings attending the catamenial discharge, with sharp, cutting pains ; pulse seventy, natural as to force and fulness. Continue rest in recumbent posture, light diet.

℞ Gum Opii ii grs
Sacch Saturni vi grs
Syrup Simp q s M.

Pillulae No. viii are to be taken every two hours till pains suspend; xx drops Spts. Nit. Dul. in water procenata.

22nd—Better; hæmorrhage and pain suspended about 5 P. M. Thinks she lost one and a half pints to one quart of blood, which was probably not far from the truth. Gave the same general directions and instructions as before.

Dec. 22nd—Was summoned at 9 o'clock P. M. Intimations of approaching labor by slight cutting pains; external parts relaxing, moist; no dilatation of the os uteri; no distinct labor pain. Seated by the bed-side of the patient, she reclining on her back, at 10 P. M., with the above condition, presenting in two minutes an intense pain, accompanied by copious flooding, announced labor rapidly progressing. Os uteri very dilutable; placenta presenting, entirely occluding the os membranes, not approachable by reason of it; liquor amuni not evacuated. Proceeded at once to detach the placenta fully. Within four minutes from the first pain, a most vigorous one came, during which the entire placenta was delivered, also a part of the waters discharged, and the head of the child was brought low down in a natural position. A third promptly followed, which completely delivered a small female child (weight five pounds) and the residue of the liquor amnii. The hæmorrhage ceased with the separation of the placenta. The child was pale—apparently dead. By persevering efforts of the usual means of resuscitation, in nine minutes it gave a spasmodic twitch of the left leg; in ten, a gasp; these were repeated, and in fifteen minutes respiration was fully established. The child was feeble and anæmic the first two years, and grew very little. Since then, had grown pretty well; is now, 1861, rather small for her years and robust. The mother made a good recovery, though somewhat enfeebled by the flooding, and has since given birth to a son without complication.

Case 5th—Dec. 29th, 1860, was called at 5 P. M., to C—— T——t, German, married; a stranger to me. Is apparently a very stout, healthy, well developed woman. Her fourth pregnancy. Says she has not felt right any of the time (this is her expression) low down in her belly, and you will find I am not right, Dr. Examination—Parts relaxing well; capacious, above the average; os uteri not dilated. Labor progressed with rapidity. At five minutes before 6 P. M., found the os dilating rapidly, the peculiar feeling of the placenta communicated to the finger. Quick as thought came a most agonizing pain, attended by a great gush of blood. The placenta and liquor amnii following the latter in large quantity. Am confident there was no hæmorrhage of any amount subsequent to the expulsion of the placenta. The pain continued till a small male child was expelled. The woman made an excellent recovery. The child grew slowly for a time, but now, Oct., 1861, is fully an average for health and strength.

